# Uptake and biotransformation of 2,2′,4,4′-tetrabromodiphenyl ether (BDE-47) in four marine microalgae species

**DOI:** 10.1038/srep44263

**Published:** 2017-03-13

**Authors:** Beverly H. K. Po, Ka-Lok Ho, Michael H. W. Lam, John P. Giesy, Jill M. Y. Chiu

**Affiliations:** 1 Department of Biology, Hong Kong Baptist University, Hong Kong; 2 School of Biological Sciences, University of Hong Kong, Hong Kong; 3 Department of Biology and Chemistry, City University of Hong Kong, Hong Kong; 4 Toxicology Centre, University of Saskatchewan, Saskatoon, SK S7N 5B3, Canada

## Abstract

Hydroxylated- and methoxylated- polybrominated diphenyl ethers (OH-PBDEs and MeO-PBDEs) are more toxic than PBDEs and occur widely in the marine environment, and yet their origins remain controversial. In this study, four species of microalgae (*Isochrysis galbana, Prorocentrum minimum, Skeletonema grethae* and *Thalassiosira pseudonana*) were exposed to BDE-47, which is synthetic and is the predominant congener of PBDEs in the environment. By chemical analysis after incubation of 2 to 6 days, the efficiency of uptake of BDE-47 and, more importantly, the potential of undergoing biotransformation to form OH-PBDEs and MeO-PBDEs by the microalgae were investigated. Growth rates of these axenic microalgae were not affected upon exposure to environmentally relevant concentrations (0.2–20 μg BDE-47 L^−1^), and accumulation ranged from 0.772 ± 0.092 μg BDE-47 g^−1^ lipid to 215 ± 54 μg BDE-47 g^−1^ lipid within 2 days. Debromination of BDE-47 and formation of BDE-28 occurred in all microalgae species (0.01 to 0.87%), but biotransformation to OH-PBDEs was only found in *I. galbana* upon exposure to extremely high concentration. The results of this study showed that biotransformation of microalgae species is unlikely an explanation for the OH-PBDEs and MeO-PBDEs found in the marine environment.

Due to their wide use as brominated flame retardants, polybrominated diphenyl ethers (PBDEs) have emerged as ubiquitous environmental contaminants. PBDEs and their analogs, hydroxylated- and methoxylated-PBDEs (OH-PBDEs and MeO-PBDEs), have been recorded in various marine biota ranging from cyanobacteria^1^, macroalgae^1^,^2^, bivalves^2^,^3^, fish^4^ to cetaceans^5^. They also have a wide geographic distribution^1^,^2^,^3^,^6^,^7^,^8^,^9^,^10^,^11^,^12^. Despite the fact that there is no known anthropogenic source of these analogues^13^, great environmental concentrations have been reported. Total MeO-PBDEs of 3.76 μg g^−1^ wet mass was found in the blubber of pygmy sperm whale^14^. In environmental samples, including the red macroalga^1^, mollusks^9^, fish^4^,^15^, as well as seals and their pups^6^, OH-PBDEs generally co-exist with MeO-PBDEs. The most commonly found congeners of these two groups of PBDE analogs are 6-MeO-BDE-47 and 6-OH-BDE-47, respectively^5^,^10^,^11^,^12^,^15^. Known effects of OH-PBDEs included developmental neurotoxicity, as seen in zebrafish associated with oxidative stress and potentially disrupted cholinergic system^16^; genotoxicity, as shown by ROS induction in chicken DT40 cell lines^17^; and endocrine disruption, as demonstrated in mouse reporter gene activity assay^18^ and rat *in vivo* experiment^19^. In general, OH-PBDEs tend to be more potent than MeO-PBDEs and PBDEs^19^,^20^,^21^. For instance, 6-OH-BDE-47 has been found to cause dysregulation of calcium ion in cortical neurons by activation of ryanodine receptors (RyR) more readily than BDE-47^20^. 6-OH-BDE-47 also induced a greater amount of ROS and caused more damage to DNA in human hepatoma cell line (HepG2) when compared with 6-MeO-BDE-47^22^.

Despite the higher toxicity comparing with PBDEs and widespread occurrence in the marine environment, OH-PBDEs and MeO-PBDEs have their origins being uncertain and controversial. Some studies suggested that they could be naturally synthesized, while others suggested that they could be products of biotransformation from synthetic PBDEs^23^. It has long been known that natural biogenic processes in the marine environment commonly produce a variety of organohalogen compounds^24^. In particular, marine sponges are known to be able to produce various organohalogens including MeO-PBDEs, either by their own tissue or by the associated cyanobacteria within them^8^,^25^. The great diversity of brominated organic compounds existing in the wild tends to support natural origins of these compounds because there is a lack of relevant PBDE precursors. Industrial processes would not have produced, or led to formation of, all of the discovered brominated compounds. Natural origins of MeO-PBDEs are further supported by the identification of related radiocarbon compounds from True beak’s whales^26^ and in sponge-cyanobacteria associations^27^, since radiocarbon could only be formed in nature but not in industrial processes. Disproval of biotransformation as the likely origin was also provided by the temporal trends of 6-MeO-BDE-47 and 2′-MeO-BDE-68 in pike from Swedish waters from 1967 to 2000, which were different from the trend of PBDEs production. Greatest concentrations of the MeO-PBDEs were detected in fish collected before 1970 while concentrations of PBDEs showed an increasing trend only up to the mid-1980s^28^.

Alternatively, results of other studies have shown the possibility of biotransformation of PBDEs to OH-PBDEs and MeO-PBDEs. For instance, human CYP2B6 has been found to be able to transform anthropogenic BDE-47 into 6 congeners of OH-PBDEs^29^. Likewise, *in vivo* study using mice dosed with DE-71 (a commercial mixture of PBDEs) have also found OH-PBDEs as metabolites in blood plasma^30^, and seven congeners of MeO-PBDEs have been detected as metabolites in blood and liver of rainbow trout (*Oncorhynchus mykiss)* after exposure to deca-PBDEs^31^. It has been suggested that bacterial *O-*methylation of PBDEs, or the OH-PBDEs from hepatic metabolism of PBDEs, might explain the formation of MeO-PBDEs^32^ because such a mechanism was the major route of biodegradation of phenols in the environment^33^. In addition, there could be interconversion between OH-PBDEs and MeO-PBDEs. For instance, Japanese medaka has been shown to be able to transform OH-PBDEs to MeO-PBDEs and vice versa, and that demethylation of MeO-PBDEs took place, at a faster rate than hydroxylation of PBDEs, to form OH-PBDEs^34^. Since such biotransformation should result in lesser concentrations of OH-PBDEs and MeO-PBDEs and since biogenic production of these analogs has only been found in a few marine species, the high concentrations of OH-PBDEs and MeO-PBDEs in animals at high trophic level is difficult to explain.

It has been suggested that an unidentified source of planktonic organisms with high turn-over rates could be responsible for the high concentrations of OH-PBDEs and MeO-PBDES found in the ocean, because the enriched ^14^C radiocarbon accumulated along the trophic level could only be acquired from surface water rather than from local hydrodynamics exposed by sessile marine sponges as suggested by many researchers^27^. Phytoplankton (or microalgae), which are abundant in surface waters and have a large surface to volume ratio, can be expected to accumulate PBDEs from water. They also play an important role in bioaccumulation of organic pollutants along the aquatic food chain. Despite this, uptake of PBDEs by microalgae has only been reported by a single study which investigated the efficiency of removal of PBDEs by freshwater microalgae in sewage treatment effluents^35^. The mechanism of passive uptake of POPs into microalgae can be described by a two-compartment model – immediate adsorption onto cell wall of the microalga followed by absorption (diffusion into the cell matrix with a mechanism similar to partitioning)^36^,^37^. However, whether microalgae could metabolize PBDEs and transform them into OH-PBDEs and MeO-PBDEs remain unknown. The significant correlations found between alkenones (a phytoplankton biomarker) and OH-PBDEs and MeO-PBDEs in surface sediment might suggest coccolithophorid species is a production source of 6-OH-BDE-47 and 6-MeO-BDE-47^38^.

This study was conducted to determine whether microalgae could synthesize OH-PBDEs and MeO-PBDEs by a) *de novo* production or b) biotransformation of PBDEs acquired from the environment. Four species of marine microalgae from different taxonomic groups were selected for experiment, including the Haptophyceae *Isochrysis galbana*, the Dinophyceae *Prorocentrum minimum*, the Bacillariophyceae *Skeletonema grethae* and the Bacillariophyceae *Thalassiosira pseudonana*. The uptake efficiencies of BDE-47 in these four different microalgae were also determined. To the best of our knowledge, this is the first attempt to investigate whether microalgae, as primary producers in the aquatic environment, could be a source of OH-PBDEs and MeO-PBDEs (from *de novo* synthesis and/or biotransformation of anthropogenic PBDEs) in the marine environment.

## Results

### Growth of microalgae exposed to BDE-47

**Figure 1:**
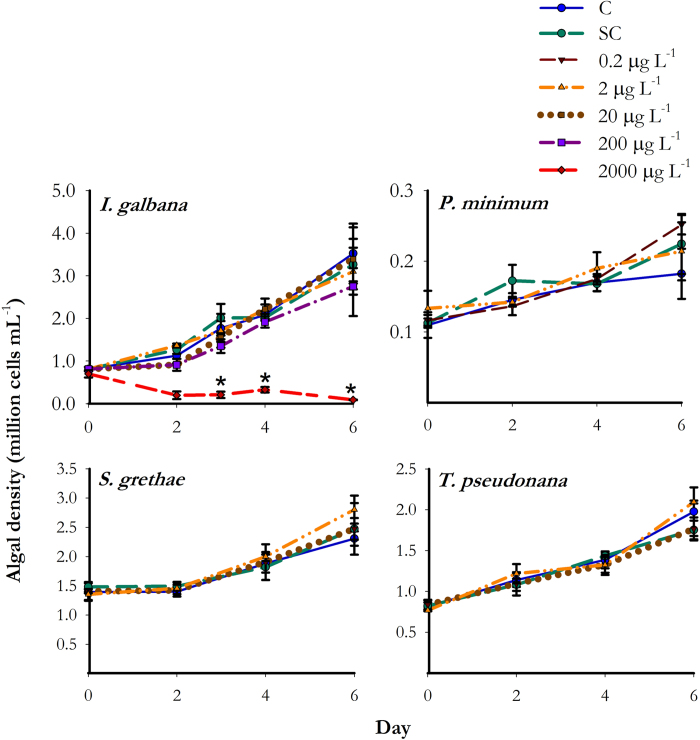
Algal density of *I. galbana, P. minimum, S. grethae*, and *T. pseudonana* over time upon exposure to BDE-47 at concentrations of 0.2, 2, 20, 200 and 2000 μg L^−1^. Microalgae were cultured using F/2 medium prepared with autoclaved filtered seawater and maintained in environmental chamber (14:10 light/dark cycle; 25 °C) in culture flasks (n = 3). Error bars showing the standard deviation. An asterisk (*) is placed adjacent to the plot where there was significant difference in algal growth when compared to control on the same time point (Holm-Sidak test, *p* < 0.05). C: Control; SC: Solvent control (0.01% DMSO).

Compared with the respective controls, similar growth with actively dividing stage were found in all species upon exposure to BDE-47 from day 0 to day 6 (Fig. 1), except negative growth was found in *I. galbana* on day 3 (F_5,2_ = 18.920, *p* < 0.001), day 4 (F_5,2_ = 87.115, *p* < 0.001), and day 6 (F_5,2_ = 14.895, *p* < 0.001) upon exposure to extreme high concentration of BDE-47 (2000 μg L^−1^).

### Uptake and bioaccumulation of BDE-47 into microalgae

**Figure 2:**
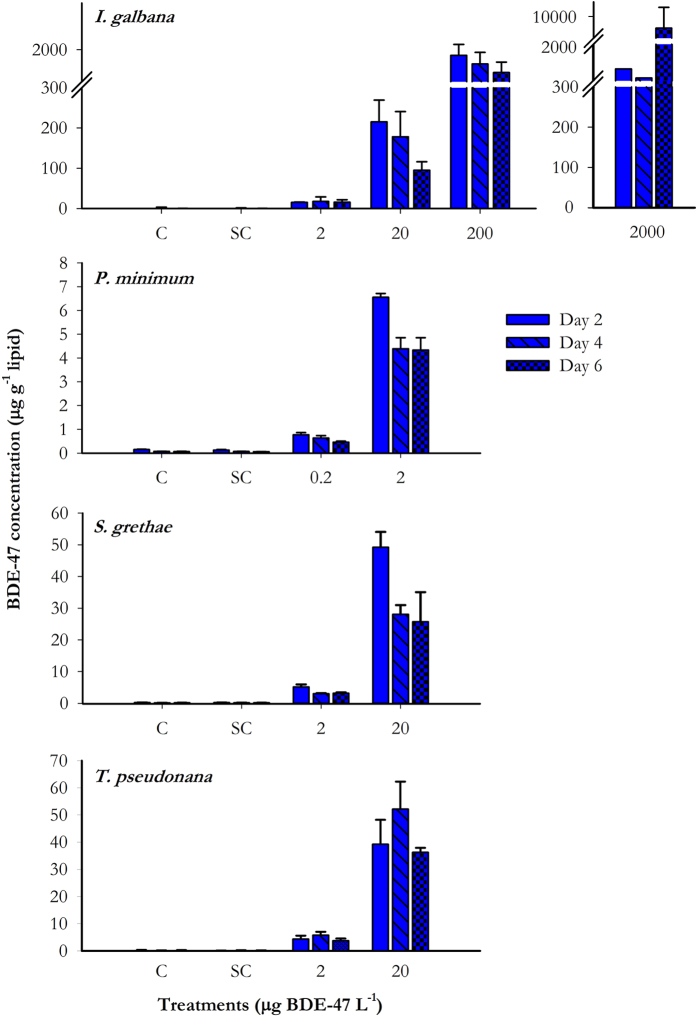
Concentrations of BDE-47 (±SD) in *I. galbana, P. minimum, S. grethae*, and *T. pseudonana* after exposure to BDE-47 at 0.2 to 2000 μg L^−1^ for 2, 4 and 6 days. N = 3. Error bars showing the standard deviations. C: Control; SC: Solvent control (0.01% DMSO).

In all species tested, uptake of BDE-47 was positively related to the exposure concentrations. For example, uptake was 5 μg g^−1^ lipid in the 2 μg L^−1^ treatment and 50 μg g^−1^ lipid in the 20 μg L^−1^ treatment in *S. grethae* on day 2 (Fig. 2). It was only in *I. galbana* exposed to 2000 μg L^−1^ where uptake concentration was not as expected due to the lack of intact cells collected for measurement. Concentrations of BDE-47 in all species decreased from day 2 to day 6. As the algal cells multiplied and biomass increased from day 2 to day 6, the lipid content was likely to increase. If the total uptake amount of BDE-47 reached the maximum by day 2, increases in biomass and lipid content on subsequent days would result in reduced lipid-normalized concentration in algal cells. The background concentration of BDE-47 in filtered seawater used in this study was 0.087 ± 0.023 μg L^−1^, whereas targeted OH-PBDEs and MeO-PBDEs were not detected.

The percentage of BDE-47 accumulated was greatest in *I. galbana*, which ranged from 56 ± 13% to 76 ± 19% excluding the extreme great concentration (Table 1). Only species (F_3,32_ = 62.179, *p* < 0.001), but not number of days of exposure (F_2,32_ = 0.741, *p* = 0.485), had a significant effect on the uptake percentages when exposed to 2 μg BDE-47 L^−1^ (Two-way ANOVA). The results of the Holm-Sidak test showed that besides *S. grethae* and *T. pseudonana*, other species exhibited different uptake percentages (*p < *0.05).

The log of the lipid-normalized bioaccumulation factors (log BAFs) ranged from 3.87 ± 0.01, 3.40 ± 0.10, 3.35 ± 0.09, and 3.26 ± 0.13 for *I. galbana, P. minimum, S. grethae*, and *T. pseudonana*, respectively. Log BAF of *I. galbana* was significantly greater than all other algal species (one-way ANOVA, F_3,8_ = 33.505, *p *<* *0.001).

### Biotransformation of BDE-47 into PBDE analogs

**Figure 3:**
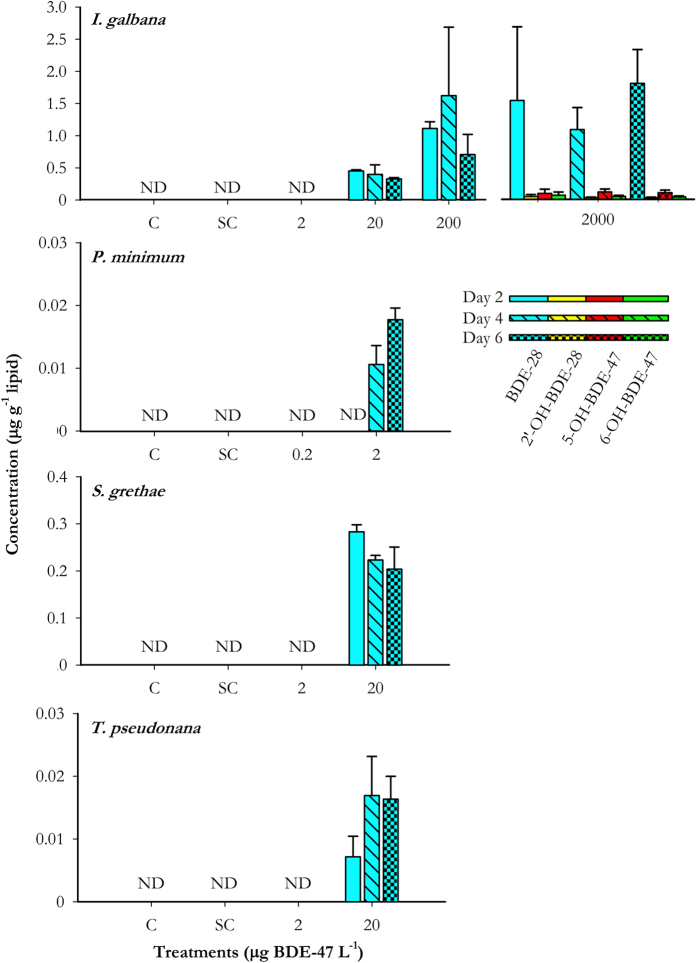
Concentrations of PBDE metabolites (±SD) in *I. galbana, P. minimum, S. grethae*, and *T. pseudonana* after exposure to BDE-47 for 2, 4 and 6 days. N = 3. Error bars showing the standard deviation. C: Control; SC: Solvent control (0.01% DMSO); ND: Not detectable.

While all of the targeted analysts besides BDE-47 were absent in the BDE-47 stock used, BDE-28 was found in all four microalgae species after exposure to the greater treatment concentrations of BDE-47 (Fig. 3). The greatest conversion percentages to BDE-28 was found in *S. grethae* (0.58–0.87%), followed by *I. galbana* (0.22–0.36%) and *P. minimum* (0.24–0.45%). The least conversion occurred in *T. pseudonana* (0.01–0.04%) (Table 2). 2′-OH-BDE-28, 5-OH-BDE-47, and 6-OH-BDE-47 were only found in *I. galbana* exposed to 2000 μg BDE-47 L^−1^. Concentrations of OH-PBDEs were small when compared with BDE-28. Conversion percentages of OH-PBDEs were about 0.003%, 0.012%, and 0.005% for 2′-OH-BDE-28, 5-OH-BDE-47, and 6-OH-BDE-47 respectively (Table 3). MeO-PBDEs were not detected in any of the samples.

## Discussion

The results from this study show that BDE-47 can be readily taken up by all four species of microalgae, either by adsorption and absorption. In just two days, uptake efficiencies of BDE-47 into microalgae had reached 76 ± 19% for the flagellates *I. galbana* and 68 ± 6% for the dinoflagellates *P. minimum*, while the diatoms *S. grethae* and *T. pseudonana* exhibited lesser uptake efficiencies (ca. 31%). This result was consistent with the finding that bioaccumulation factors (BAF) of PBDEs by *P. minimum* being greater than that of *Thalassiosira* species^39^. This could have been due to polysaccharides in cell walls of diatoms^40^ which are produced in larger amounts than by the other phytoplankton species^41^. The hydrophilic polysaccharides could reduce the amount of lipophilic BDE-47 adsorbed to cells, thus resulting in lesser bioaccumulation. There is only limited information on concentrations of PBDEs in marine microalgae, except that 0.023 μg BDE-47 g^−1^ lipid was reported in *Thalassiosira* species from the South Pole^42^. The concentration of BDE-47 accumulated (4.7 μg g^−1^ lipid) by *T. pseudonana* in this experiment was 100-fold greater than the reported concentration by Chiuchiolo *et al*.^42^. However, a more appropriate comparison with contaminated area is not possible since no other studies have reported concentration of PBDEs in microalgae from marine environments (also reviewed in Kosek *et al*.^43^). The log BAFs for BDE-99 obtained during a 2-day incubation for *P. minimum* and *Thalassiosira weissflogii* were approximately 8.5^39^, which means BAFs were several orders of magnitude greater than those observed in this study for BDE-47.

Both 6-MeO-BDE-47 and 6-OH-BDE-47 were absent from the controls of all species used in this study, showing that these four species of microalgae could not produce these PBDE analogs naturally in the absence of PBDE as the precursor. Neither OH-PBDEs nor MeO-PBDE was detectable in filtered seawater used for the experiments. The result of this study therefore did not support the self-production or biotransformation of 6-MeO-BDE-47 by phytoplankton. However, 2′-MeO-BDE-68 and 6-MeO-BDE-47 were detected at pg g^−1^ dry mass concentrations in axenic cultures of *Chaetoceros curvisetus, Prorocentrum donhaiense*, and *Emiliania huxleyi*^44^, which suggested that biosynthesis may be possible, at least in some species. The difference in findings might be due to the differences in algal species, experimental protocol (in which seawater was only filtered through 0.45 μm, allowing passing of certain bacteria that may perform biotransformation^45^) and source of seawater (in which the background levels of PBDEs and their metabolites in the seawater obtained from the East China Sea were not analyzed in their study). Concentrations of PBDEs in seawater in the East China Sea has not been measured previously^46^, but the same MeO-PBDE congeners in the microalgae cultures in Fan *et al*.^44^ were also found at concentrations of 0.001–0.022 μg g^−1^ lipid in shellfish from the same area^47^. Although this might not directly confirm the presence of MeO-PBDEs or PBDEs in seawater because these pollutants might be transported through trophic transfer, their presence in natural seawater used for algal culture in their study cannot be ruled out. Mean concentrations of 2′-MeO-BDE-68 and 6-MeO-BDE-47 in seawater in Queensland were estimated to be 41 and 58 pg L^−1^ (by passive samplers)^48^, and the presence of MeO-PBDEs in water cannot be ruled out because the concentration might be too low to be detected by solvent-extraction.

Since algal cells were actively dividing during incubation, the presence of BDE-28 in some of the cultures would support the assumption that BDE-47 had been available for metabolism within algal cells. The detection of BDE-28 in cells exposed to greater concentrations of BDE-47 (1 μg L^−1^ for *P. minimum* and 10 μg L^−1^ for others) demonstrated that debromination had taken place during the incubation. BDE-28 was only detected on day 4 and 6 in *P. minimum* while it occurred at all sampling time points for the other three species. In nature, debromination is a common process to breakdown PBDEs, and light could accelerate debromination rate^49^. The tetra-brominated BDE-47 itself in the environment was assumed to be formed by debromination from more brominated congeners, such as the decabrominated BDE-209, derived from flame retardants. However, in both field and laboratory situations, light must be available to support growth and metabolism of microalgae and as such the possibility of photo-debromination could not be eliminated. A recent study demonstrated that tolerant freshwater microalgae isolated from wastewater treatment plants can degrade 68–86% of tetra-brominated BDE-47 in 7 days^35^, suggesting that removal of BDE-47 by marine microalgae could also be possible.

The presence of OH-PBDEs at the extreme high concentration of BDE-47 (2000 μg L^−1^) in *I. galbana* indicated potential capability for this species to metabolize PBDEs into OH-PBDEs by biotransformation. The metabolite 6-OH-BDE-47 has also been found in bile of crucian carp exposed to the hexa-brominated BDE-153^50^. Although the tetra-brominated 5-OH-BDE-47 and tri-brominated 2′-OH-BDE-28 were not congeners targeted in their study, OH-tetra-BDE and OH-tri-BDE with unknown structures were shown in their GC-MS data^50^. Hydroxylation of PBDEs could be catalyzed by enzymes of the CYP family in mammals^29^, and genes encoding for CYPs have also been found in single-celled diatoms^51^. Nonetheless, further mechanistic studies such as pathway analysis are necessary to explain why biotransformation only occurred at such high concentration and when growth rate was inhibited.

The finding of OH-PBDEs in this study must be interpreted with caution due to very low concentrations of metabolite found. In contrast to this study, it was found that common sole probably produced 4′-OH-BDE-49 and 4′-OH-BDE-101 as metabolite products after PBDE exposure, while 6-OH-BDE-47 in the fish was mainly of biogenic nature^52^. Nonetheless, the fact that no OH-PBDE was found in both controls and treatments at lower concentrations showed that OH-PBDEs were not produced by biogenesis in *I. galbana*. The majority of OH-PBDEs found in biota were believed to be transformed by demethylation from MeO-PBDEs within the organisms^23^,^34^,^53^. However, no MeO-PBDEs could be detected in any microalgae species in this study. Direct biotransformation (hydroxylation) from BDE-47 to OH-BDE-47 with debromination before or after the transformation might therefore be the possible mechanism. Hydroxylation is generally the first step of xenobiotic detoxification, and it was suggested to serve as a mechanism for PBDE removal in tolerant microalgae^35^. Yet, one cannot rule out the possibility that MeO-PBDEs, as intermediate metabolite, became too low to be detected since the biotransformation from MeO-PBDEs to OH-PBDEs was very efficient. The extreme high BDE-47 concentration used in this experiment shed light on the biological response at unrealistically great concentration. Notably, strong growth inhibition was found at the 2000 μg L^−1^ treatment within 2 days. The great BDE-47 concentration might have triggered certain atypical cell mechanism to produce OH-PBDEs, and thereby exerted toxic effects and inhibited cell growth. It has been shown that *ortho*-tetra-BDE radical was formed readily by photolysis of BDE-47, and that the formation of hydroxyl radical (•OH) was essential for the photo-formation of OH-PBDEs from PBDEs^21^. If the microalgae had formed hydroxyl radical during the exposure, this might as well be the cause of the observed growth inhibition.

In conclusion, 0.2–20 μg BDE-47 L^−1^ did not inhibit growth of the four microalgae species studied within 6 days, but biotransformation of BDE-47 to BDE-28 was clearly evident in all species. Only under extreme high BDE-47 concentration that small amount of OH-PBDEs metabolites (i.e. 2′-OH-BDE-28, 5-OH-BDE-47, and 6-OH-BDE-47) were produced in *I. galbana* associated with growth inhibition. Besides, there was no natural production of the targeted OH-PBDEs nor MeO-PBDEs found in the microalgae species tested here. It should be noted that these findings were limited to the exposure conditions and analytical methods used in this study. However, with the exposure to environmentally-relevant concentration of BDE-47, this study has found that it is unlikely for the four microalgae species to produce the high concentrations of OH-PBDEs and MeO-PBDEs observed in the marine environment by either *de novo* production or biotransformation.

## Methods

### Culture and spiking of BDE-47

Seawater used was collected from the Swire Institute of Marine Science at the Cape d’Aguilar Marine Reserve of Hong Kong Island. All glassware was rinsed with acetone before ashing at 500 °C for 7 hours to remove any organic contaminant. The four marine microalgae *I. galbana* (clone T-ISO), *P. minimum* (CCMP 2780), *S. grethae* (CCAP 1077/3, formerly listed as *Skeletonema costatum*) and *T. pseudonana* (CCMP 1335) was obtained from the axenic culture from the City University of Hong Kong and the laboratory of Prof. KMY Leung of School of Biological Sciences, University of Hong Kong. All microalgae have been cultured in the laboratory for over a year. The microalgae were cultured using F/2 medium by Guillard^54^ prepared with autoclaved filtered seawater (FSW). Sodium metasilicate was added to *S. grethae* and *T. pseudonana* to achieve concentration at 1.06 × 10^–4^ M. The glass bottles carrying the microalgae were aerated with 0.22 μm filtered air and maintained in environmental chamber (14:10 light/dark cycle; 25 °C). Light was provided by white fluorescent tubes and 22 W white light lamps (intensity of approximately 2700 LUX). Algal growth rate was estimated by algal density determination with a haemocytometer.

Stock solutions of BDE-47 (Chem Service Inc.) at 2 × 10^6^, 2 × 10^5^, 2 × 10^4^, 2 × 10^3^ μg L^−1^ BDE-47 stocks were prepared with DMSO by serial dilution from a stock of 10^7^ μg L^−1^. Triplicates of BDE-47 treatments with microalgae in Erlenmeyer flasks were set up. During treatment, microalgae culture which has reached log-phase was sub-cultured into new F/2 medium to reach 1 × 10^5^ cells mL^−1^ (for *P. minimum*) and 1 × 10^6^ cells mL^−1^ for all the other three species. BDE-47 stock solutions were added to the culture flasks to produce exposure concentrations of 2000, 200, 20, 2 or 0.2 μg L^−1^ in 300 mL of microalgae culture. In these culture media, 30 μL of BDE-47 stock solutions (or DMSO for the solvent control) was used to provide the same solvent concentrations (0.01%, v/v) in all treatments. The culture flasks were swirled twice per day during exposure. On day 2, 4 and 6 after inoculation, 50 mL of microalgae were collected from each flask and subject to centrifugation (3000 rpm, 5 min., 4 °C). The algal cells were re-suspended with FSW and then centrifuged with 15 mL glass tubes. The samples were then freeze-dried, weighed for dry weights, and stored at −20 °C before chemical analysis.

The growth rate (doubling time) and the tolerance to BDE-47 and DMSO for each species found from the literature and preliminary tests under laboratory conditions are summarized in Table 4. Result of a preliminary experiment showed that algal absorption of BDE-47 was below 60% within 1 day. As a result, algae were collected on day 2, 4 and 6 in subsequent experiments, to ensure that the algal cells can multiply at least once and also that significant amount of BDE-47 could be up-taken by the microalgae. Since log phase was reached by day 6, algal cells should have completed their life cycle, and biotransformation, if any, should have occurred. The concentration of the solvent DMSO at 0.01% is below the NOEC values (≥0.058%)^55^. Except for *P. minimum* which ordinarily had a lower density at log-phase and had shown growth inhibition at 2 μg BDE-47 L^−1^, the nominal exposure concentrations for the other 3 species were 2 μg BDE-47 L^−1^ (the greatest environmental concentration reported) and a magnitude greater at 20 μg BDE-47 L^−1^ (greater confidence of measurement above detection limit). Exposure concentration for *P. minimum* was 0.2 and 2 μg BDE-47 L^−1^. 200 and 2000 μg BDE-47 L^−1^ were used in an additional experiment for *I. galbana* to test for response under extreme high concentrations.

### Chemical analysis

The background level of PBDEs in FSW was measured in four replicates. Organic chemicals were extracted by same volume of hexane for three times in a separation funnel. The extract was dried and then re-dissolved in 100 μL of hexane before measurement.

**Figure 4:**
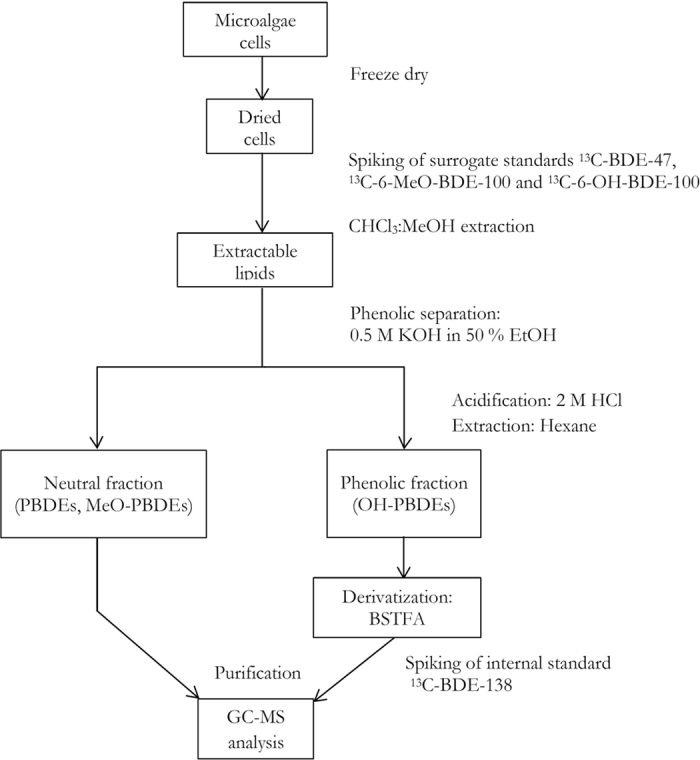
Procedures of chemical pre-treatment for analysis of PBDEs and their derivatives prior to GC-MS analysis.

For microalgae, the extraction and clean-up were based on Wang *et al*.^56^ with modifications. The flow chart in Fig. 4 shows the procedures for the chemical analysis of microalgae. Firstly, samples were spiked with surrogate standard of ^13^C-BDE-47, ^13^C-6-MeO-BDE-100 and ^13^C-6-OH-BDE-100 (Wellington Laboratories, Guelph, ON, Canada) in acetone. For lipid extraction, 5 mL of chloroform/methanol (1:1) was added and homogenized with algal cells by sonication at 40 °C for 30 min followed by centrifugation for three times. The solvent was dried by a stream of nitrogen gas, and the lipid weight was determined gravimetrically. After re-dissolving by 4 mL of hexane, phenolic phase was separated by adding 2 mL of 0.5 M potassium hydroxide in 50% ethanol and then mixed by vortex. The upper neutral layer was transferred to new tubes and extracted once again by hexane. The phenolic fraction that partitioned into the aqueous layer was then acidified by 2 mL of 2 M hydrochloric acid followed by two extractions to 4 mL of hexane/MTBE (9:1; v/v). Both fractions were then blow dried by nitrogen before purification.

The neutral fraction was purified by passing through a column with acidified silica gel, aluminum oxide and anhydrous sodium sulphate. Chemicals were eluted by 2 mL of hexane followed by 2 mL of DCM. For purification of the phenolic fraction, a column of fluorosil and sodium sulphate was used. Chemicals were eluted by 2 mL of hexane/DCM (1:1; v/v) and 2 mL of DCM. The eluates of phenolic fraction were then incubated with 50 μL of BSTFA (Supelco™ Analytical, USA; 99.6%) at 70 °C water bath for 1 hour for derivatization. The two fractions were injected separately into GC-MS with ^13^C-BDE-138 spiked as internal standard before GC-MS injection at concentration of 200 μg L^−1^ for both fractions.

### Instrumentation

Two PBDE congeners (BDE-47 and BDE-28), three MeO-PBDEs congeners (2′-MeO-BDE-28, 5-MeO-BDE-47 and 6-MeO-BDE-47) and three OH-PBDEs (2′-OH-BDE-28, 5-OH-BDE-47 and 6-OH-BDE-47) (99–100%; AccuStandard Inc., New Haven, CT, USA) were the target analytes for investigation. 5-MeO-BDE-47 and 6-MeO-BDE-47 and their hydroxylated analogs were chosen because they are the most abundant congeners of their kinds in environmental samples, while the tri-brominated 2′-MeO-BDE-28 and 2′-OH′-BDE-28 were targeted since they are possible metabolites either from debromination of their tetra-brominated MeO-PBDEs and OH-PBDEs analogs or from methoxylation or hydroxylation of BDE-28. They were quantified by gas chromatography interfaced to a mass spectrometry equipped with VF-5MS (Bruker 450 GC/320 MS) or DB-5MS (Agilent Technologies 7890A GC/5975 MSD) capillary column (30 m length, 0.25 mm ID, 0.25 μm film) with helium as the carrier gas for chromatographic separation for all target analysts. Detail of instrumentation with GC-MS was same as in Ho *et al*.^57^ using Electron Ionization (EI) mode with Selective Ion-Monitoring (SIM). The injector temperature was held at 280 °C for 41 min of run time. The column oven programme was as follow: from 60 °C (2 min) increased at a rate of 15 °C min^−1^ to 250 °C, then increased at a rate of 5 °C min^−1^ to 280 °C which was held for 10 min, and lastly increased at a rate of 30 °C min^−1^ to 290 °C and held for 10 min. The standard curves were obtained by injecting at 1 to 500 ng mL^−1^ for PBDEs and MeO-PBDEs, or 0.5 to 500 ng mL^−1^ for OH-PBDEs.

### Quality control

Procedural blanks which consisted of the three surrogate standards were used for background reduction for every 12 to 15 samples. The average recovery percentages as determined by the mass of surrogate standards in each sample was 89% (69–113%) for ^13^C-BDE-47, 77% (70–95%) for ^13^C-6-MeO-BDE-100 and 86% (71–110%) for ^13^C-6-OH-BDE-100. To confirm the purities of the BDE-47 stock used for treatment, the stock at 2 × 10^5^ μg L^−1^ was analyzed by the same method and no BDE-28, MeO-PBDEs nor OH-PBDEs could be detected.

### Calculations and statistical analysis

For uptake percentage of BDE-47, calculations were done by dividing the mass of BDE-47 detected by the nominal BDE-47 mass for the volume collected per sample:


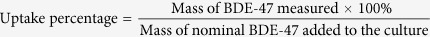


Bioaccumulation is defined as the accumulation of chemical as a result from uptake from all sources (including surrounding water), and bioaccumulation factor (BAF, L kg^−1^) is defined as the ratio of analyte in the biota and water^58^. For this study, its calculation is essentially the same as the bioconcentration factor:


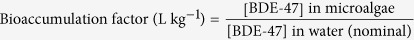


For percentage of conversion to particular metabolite, it was calculated by finding the ratio of the metabolite to the BDE-47 measured:


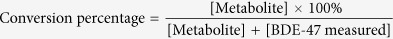


Statistical analysis was performed using SigmaPlot 11.0. One-way ANOVA was used to find difference in algal density in different treatments at the same time point. Two-way ANOVA was used to find difference in the uptake percentage (arcsin square root transformed) of BDE-47 by number of days of exposure and by species. Only the concentration which had been tested in all species (2 μg L^−1^) was subjected to test for difference among species. Data passed normality test (Shapiro-Wilk) and had equal variability (both *p* > 0.05). Holm-Sidak test was performed and each treatment was compared to the control if significant difference was found by ANOVA (*p* < 0.05).

## Additional Information

**How to cite this article:** Po, B. H. K. *et al*. Uptake and biotransformation of 2,2′,4,4′-tetrabromodiphenyl ether (BDE-47) in four marine microalgae species. *Sci. Rep.* **7**, 44263; doi: 10.1038/srep44263 (2017).

**Publisher's note:** Springer Nature remains neutral with regard to jurisdictional claims in published maps and institutional affiliations.

## Figures and Tables

**Table 1:** Uptake percentages (±SD; n = 3; corrected to the nearest 1%) of BDE-47 from nominal concentrations of 0.2, 2, 20, 200, and 2000 μg L^−1^ in the phytoplankton species *I. galbana, P. minimum, S. grethae*, and *T. pseudonana*.

	Uptake percentage			
	*I. galbana*	*P. minimum*	*S. grethae*	*T. pseudonana*
Day 2				
0.2 μg L^−1^	—	68 ± 6%	—	—
2 μg L^−1^	65 ± 7%^A^	50 ± 4%^B^	39 ± 0%^C^	27 ± 4%^C^
20 μg L^−1^	76 ± 19%	—	36 ± 3%	29 ± 3%
200 μg L^−1^	71 ± 8%	—	—	—
2000 μg L^−1^	4 ± 2%	—	—	—
Day 4				
0.2 μg L^−1^	—	59 ± 1%	—	—
2 μg L^−1^	69 ± 14%^A^	43 ± 5%^B^	31 ± 4%^C^	31 ± 8%^C^
20 μg L^−1^	71 ± 21%	—	27 ± 2%	28 ± 12%
200 μg L^−1^	58 ± 17%	—	—	—
2000 μg L^−1^	5 ± 3%	—	—	—
Day 6				
0.2 μg L^−1^	—	56 ± 2%	—	—
2 μg L^−1^	69 ± 9%^A^	49 ± 5%^B^	31 ± 2%^C^	37 ± 2%^C^
20 μg L^−1^	56 ± 13%	—	27 ± 4%	34 ± 3%
200 μg L^−1^	64 ± 13%	—	—	—
2000 μg L^−1^	105 ± 8%	—	—	—

Results with significant difference (Two-way ANOVA, *p* < 0.05) among microalgae species are represented by different alphabets.

**Table 2:** Conversion percentages (±SD; n = 3; corrected to the nearest 0.01%) from BDE-47 to BDE-28 in *I. galbana, P. minimum, S. grethae*, and *T. pseudonana* after BDE-47 treatment for 2, 4 and 6 days.

	*I. galbana*	*P. minimum*	*S. grethae*	*T. pseudonana*
Day 2				
0.2 μg L^−1^	—	ND	—	—
2** **μg L^−1^	ND	0.24 ± 0.04%	ND	ND
20** **μg L^−1^	0.22 ± 0.07%	—	0.58 ± 0.06%	0.01 ± 0.01%
2000 μg L^−1^	0.06 ± 0.01%	—	—	—
Day 4				
0.2 μg L^−1^	—	ND	—	—
2** **μg L^−1^	ND	0.24 ± 0.04%	ND	ND
20** **μg L^−1^	0.22 ± 0.03%	—	0.80 ± 0.08%	0.03 ± 0.02%
2000 μg L^−1^	0.11 ± 0.07%	—	—	—
Day 6				
0.2 μg L^−1^	—	ND	—	—
2** **μg L^−1^	ND	0.45 ± 0.02%	ND	ND
20** **μg L^−1^	0.36 ± 0.07%	—	0.87 ± 0.26%	0.04 ± 0.01%
2000 μg L^−1^	0.06 ± 0.01%	—	—	—

ND: not detectable; —: not available.

**Table 3:** Conversion percentages (±SD; n = 3; corrected to the nearest 0.001%) for the OH-PBDE metabolites from *I. galbana* exposed to 2000 μg BDE-47 L^−1^.

Metabolites	Day 2	Day 4	Day 6
2′-OH-BDE-28	0.003 ± 0.000%	0.003 ± 0.000%	0.000 ± 0.000%
5-OH-BDE-47	0.007 ± 0.001%	0.012 ± 0.002%	0.001 ± 0.000%
6-OH-BDE-47	0.004 ± 0.001%	0.005 ± 0.001%	0.000 ± 0.000%

The same congeners were not detected in *P. minimum, S. grethae*, and *T. pseudonana* in the same experiment.

**Table 4:** Growth rates, resistance to the solvent DMSO and known resistance to BDE-47 of the four species of microalgae in the experiment.

	*Isochrysis galbana*	*Prorocentrum minimum*	*Skeletonema grethae*	*Thalassiosira pseudonana*
Optimal culture temperature	25 °C	20 °C	22 °C	22 °C
Approximate doubling time at log-phase	1.0/day	0.3/day	0.5/day	0.2/day
Time to reach log phase	5–7 days	7–9 days	5–7 days	5–7 days
Algal density at stationary phase	5 × 10^6^ cells mL^−1^	3 × 10^5^ cells mL^−1^	4 × 10^6^ cells mL^−1^	5 × 10^6^ cells mL^−1^
DMSO toxicity (NOEC value)	0.058% Ref. 55	0.700% Ref. 55	1.100% Ref. 55	N/A
BDE-47 toxicity (literature information or lab test)	NOEC = 2.53 μg L^−1^LOEC = 5.06 μg L^−1^IC_50_ = 25.7 μg L^−1^Ref. 59	Growth inhibition by 20% at 2 μg L^−1^	NOEC = 6.6 μg L^−1^ EC_50 = _70 μg L^−1^ Ref. 60	Growth inhibition by 10% at 200 μg L^−1^
Nominal exposure concentration used (μg BDE-47 L^−1^)	(1) 2, 20, 200 (2) 2000	0.2, 2	2, 20	2, 20

NOEC: No observable effect concentration; LOEC: Lowest observed effect concentration; IC_50_: Half maximal inhibitory concentration; EC_50_: Median effect concentration. Information with no reference was obtained from preliminary experiments in laboratory for range finding.

## References

[b1] Malmvärn A., Zebuhr Y., Kautsky L., Bergman A. & Asplund L. Hydroxylated and methoxylated polybrominated diphenyl ethers and polybrominated dibenzo-p-dioxins in red alga and cyanobacteria living in the Baltic Sea. Chemosphere 72, 910–916 (2008). 18457860 10.1016/j.chemosphere.2008.03.036

[b2] Malmvärn A. . Hydroxylated and methoxylated brominated diphenyl ethers in the red algae *Ceramium tenuicorne* and blue mussels from the Baltic Sea. Environ. Sci. Technol. 39, 2990–2997 (2005). 15926543 10.1021/es0482886

[b3] Liu X., Jiao Y., Lin C., Sun K. & Zhao Y. PBDEs, hydroxylated PBDEs and methoxylated PBDEs in bivalves from Beijing markets. Chemosphere 110, 97–103 (2014). 24636323 10.1016/j.chemosphere.2014.02.019

[b4] Wang H.-S. . Exposure of Hong Kong residents to PBDEs and their structural analogues through market fish consumption. J. of Hazard. Mater. 192, 374–380 (2011). 21658843 10.1016/j.jhazmat.2011.05.036

[b5] Nomiyama K. . Anthropogenic and naturally occurring polybrominated phenolic compounds in the blood of cetaceans stranded along Japanese coastal waters. Environ. Pollut. 159, 3364–3373 (2011). 21903310 10.1016/j.envpol.2011.08.035

[b6] Weijs L. . Methoxylated PBDEs (MeO-PBDEs), hydroxylated PBDEs (HO-PBDEs) and hydroxylated PCBs (HO-PCBs) in the liver of harbor seals from the northwest Atlantic. Sci. Total Environ. 493, 606–614 (2014). 24982026 10.1016/j.scitotenv.2014.06.028

[b7] Losada S. . Biomagnification of anthropogenic and naturally-produced organobrominated compounds in a marine food web from Sydney Harbour, Australia. Environ. Int. 35, 1142–1149 (2009). 19665796 10.1016/j.envint.2009.07.008

[b8] Haraguchi K., Kato Y., Ohta C., Koga N. & Endo T. Marine sponge: A potential source for methoxylated polybrominated diphenyl ethers in the Asia-Pacific food web. J. Agr. Food Chem. 59, 13102–13109 (2011). 22034991 10.1021/jf203458r

[b9] Sun J., Liu J., Liu Y. & Jiang G. Hydroxylated and methoxylated polybrominated diphenyl ethers in mollusks from Chinese coastal areas. Chemosphere 92, 322–328 (2013). 23582706 10.1016/j.chemosphere.2013.03.042

[b10] Roszko M., Szymczyk K., Rzepkowska M. & Jerzejczak R. Preliminary study on brominated dioxins/furans and hydroxylated/methoxylated PBDEs in Baltic cod (*Gadus morhua*) liver. Comparison to the levels of analogue chlorinated co-occurring pollutants. Mar. Pollut. Bull. 96, 165–175 (2015). 26002098 10.1016/j.marpolbul.2015.05.032

[b11] Kelly B. C., Ikonomou M. G., Blair J. D. & Gobas F. A. P. C. Hydroxylated and methoxylated polybrominated diphenyl ethers in a Canadian Arctic marine food web. Environ. Sci. Technol. 42, 7069–7077 (2008). 18939528 10.1021/es801275d

[b12] Wan Y. . Origin of hydroxylated brominated diphenyl ethers: Natural compounds or man-made flame retardants? Environ. Sci. Technol. 43, 7536–7542 (2009). 19848173 10.1021/es901357u

[b13] Vetter W. Marine halogenated natural products of environmental relevance. In Reviews of Environmental Contamination and Toxicology Vol. 188 (eds Ware GeorgeW .) 1–57 (Springer New York, 2006) 17016915 10.1007/978-0-387-32964-2_1

[b14] Vetter W. . Sponge halogenated natural products found at parts-per-million levels in marine mammals. Environ. Toxicol. Chem. 21, 2014–2019 (2002). 12371475

[b15] Zhang K. . Tissue concentrations of polybrominated compounds in Chinese Sturgeon (*Acipenser sinensis*): Origin, hepatic sequestration, and maternal transfer. Environ. Sci. Technol. 44, 5781–5786 (2010). 20604581 10.1021/es100348g

[b16] Usenko C. Y., Hopkins D. C., Trumble S. J. & Bruce E. D. Hydroxylated PBDEs induce developmental arrest in zebrafish. Toxicol. Appl. Pharm. 262, 43–51 (2012). 10.1016/j.taap.2012.04.017 22546086

[b17] Ji K., Choi K., Giesy J. P., Musarrat J. & Takeda S. Genotoxicity of several polybrominated diphenyl ethers (PBDEs) and hydroxylated PBDEs, and their mechanisms of toxicity. Environ. Sci. Technol. 45, 5003–5008 (2011). 21545137 10.1021/es104344e

[b18] Hu W. . Endocrine effects of methoxylated brominated diphenyl ethers in three *in vitro* models. Mar. Pollut. Bull. 62, 2356–2361 (2011). 21930287 10.1016/j.marpolbul.2011.08.037

[b19] Liu H. . *In vitro* profiling of endocrine disrupting potency of 2,2′,4,4′-tetrabromodiphenyl ether (BDE47) and related hydroxylated analogs (HO-PBDEs). Mar. Pollut. Bull. 63, 287–296 (2011). 21737105 10.1016/j.marpolbul.2011.04.019

[b20] Kim K. H. . Para- and ortho-substitutions are key determinants of polybrominated diphenyl ether activity toward ryanodine receptors and neurotoxicity. Environ. Health Persp. 119, 519–526 (2011). 10.1289/ehp.1002728 PMC3080935 21106467

[b21] Zhao Q., Zhao H., Quan X., He X. & Chen S. Photochemical formation of hydroxylated polybrominated diphenyl ethers (OH-PBDEs) from polybrominated diphenyl ethers (PBDEs) in aqueous solution under simulated solar light irradiation. Environ. Sci. Technol. 49, 9092–9099 (2015). 26134578 10.1021/acs.est.5b01240

[b22] An J. . The cytotoxic effects of synthetic 6-hydroxylated and 6-methoxylated polybrominated diphenyl ether 47 (BDE47). Environ. Toxicol. 26, 591–599 (2011). 20549613 10.1002/tox.20582

[b23] Wiseman S. B. . Polybrominated diphenyl ethers and their hydroxylated/methoxylated analogs: Environmental sources, metabolic relationships, and relative toxicities. Mar. Pollut. Bull. 63, 179–188 (2011). 21439595 10.1016/j.marpolbul.2011.02.008

[b24] Gribble G. W. The diversity of naturally produced organohalogens. Chemosphere 52, 289–297 (2003). 12738253 10.1016/S0045-6535(03)00207-8

[b25] Unson M. D., Holland N. D. & Faulkner D. J. A brominated secondary metabolite synthesized by the cyanobacterial symbiont of a marine sponge and accumulation of the crystalline metabolite in the sponge tissue. Mar. Biol. 119, 1–11 (1994).

[b26] Teuten E. L., Xu L. & Reddy C. M. Two abundant bioaccumulated halogenated compounds are natural products. Science 307, 917–920 (2005). 15705850 10.1126/science.1106882

[b27] Guitart C. . Contemporary ^14^C radiocarbon levels of oxygenated polybrominated diphenyl ethers (O-PBDEs) isolated in sponge–cyanobacteria associations. Mar. Pollut. Bull. 62, 631–636 (2011). 21276990 10.1016/j.marpolbul.2010.12.022 PMC4876816

[b28] Kierkegaard A. . Polybrominated diphenyl ethers (PBDEs) and their methoxylated derivatives in pike from Swedish waters with emphasis on temporal trends, 1967–2000. Env. Pollut. 130, 187–198 (2004). 15158033 10.1016/j.envpol.2003.12.011

[b29] Feo M. L. . Biotransformation of BDE-47 to potentially toxic metabolites is predominantly mediated by Human CYP2B6. Environ. Health Persp. 121, 440–446 (2013). 10.1289/ehp.1205446 PMC3620761 23249762

[b30] Qiu X., Mercado-Feliciano M., Bigsby R. M. & Hites R. A. Measurement of polybrominated diphenyl ethers and metabolites in mouse plasma after exposure to a commercial pentabromodiphenyl ether mixture. Environ. Health Persp. 115, 1052–1058 (2007). 10.1289/ehp.10011 PMC1913597 17637922

[b31] Feng C. . Debrominated and methoxylated polybrominated diphenyl ether metabolites in rainbow trout (*Oncorhynchus mykiss*) after exposure to decabromodiphenyl ether. J. Environ. Sci. 22, 1425–1434 (2010). 10.1016/s1001-0742(09)60271-0 21174975

[b32] Haglund P. S., Zook D. R., Buser H. R. & Hu J. W. Identification and quantification of polybrominated diphenyl ethers and methoxy-polybrominated diphenyl ethers in Baltic biota. Environ. Sci. Technol. 31, 3281–3287 (1997).

[b33] Allard A. S., Remberger M. & Neilson A. H. Bacterial O-methylation of halogen-substituted phenols. Appl. Environ.Microb. 53, 839–845 (1987). 10.1128/aem.53.4.839-845.1987 PMC203766 3579284

[b34] Wan Y. . Interconversion of hydroxylated and methoxylated polybrominated diphenyl ethers in Japanese medaka. Environ. Sci. Technol. 44, 8729–8735 (2010). 20973477 10.1021/es102287q

[b35] Deng D. & Tam N. F. Isolation of microalgae tolerant to polybrominated diphenyl ethers (PBDEs) from wastewater treatment plants and their removal ability. Bioresource Technol. 177, 289–297 (2015). 10.1016/j.biortech.2014.11.103 25496950

[b36] Axelman J., Broman D. & Näf C. Field measurements of PCB partitioning between water and planktonic organisms: Influence of growth, particle size, and solute−solvent interactions. Environ. Sci. Technol. 31, 665–669 (1997).

[b37] Skoglund R. S., Stange K. & Swackhamer D. L. A kinetics model for predicting the accumulation of PCBs in phytoplankton. Environ. Sci. Technol. 30, 2113–2120 (1996).

[b38] Fan Y. . Spatial distributions of methoxylated and hydroxylated polybrominated diphenyl ethers in the East China Sea—A seaward increasing trend. Chemosphere 114, 247–254 (2014). 25113209 10.1016/j.chemosphere.2014.04.103

[b39] Magnusson K., Magnusson M., Östberg P., Granberg M. & Tiselius P. Bioaccumulation of ^14^C-PCB 101 and ^14^C-PBDE 99 in the marine planktonic copepod *Calanus finmarchicus* under different food regimes. Mar. Environ. Res. 63, 67–81 (2007). 16949662 10.1016/j.marenvres.2006.07.001

[b40] Kiørboe T. & Hansen J. L. S. Phytoplankton aggregate formation: observations of patterns and mechanisms of cell sticking and the significance of exopolymeric material. J. Plankton Res. 15, 993–1018 (1993).

[b41] Decho A. W. Microbial exopolymer secretions in ocean environments: Their role(s) in food webs and marine processes. Oceanogr. Mar. Biol. 28, 73–153 (1990).

[b42] Chiuchiolo A. L., Dickhut R. M., Cochran M. A. & Ducklow H. W. Persistent organic pollutants at the base of the antarctic marine food web. Environ. Sci. Technol. 38, 3551–3557 (2004). 15296304 10.1021/es0351793

[b43] Kosek K., Polkowska Ż., Żyszka B. & Lipok J. Phytoplankton communities of polar regions–Diversity depending on environmental conditions and chemical anthropopressure. J. Environ. Manage. 171, 243–259 (2016). 26846983 10.1016/j.jenvman.2016.01.026

[b44] Fan Y. . Major sources of MeO/OH-BDEs in the East China Sea elucidated from their records and phytoplankton biomarkers. Environ. Pollut. 192, 1–8 (2014). 24874793 10.1016/j.envpol.2014.04.037

[b45] Tabor P., Ohwada K. & Colwell R. Filterable marine bacteria found in the deep sea: Distribution, taxonomy, and response to starvation. Microb Ecol 7, 67–83 (1981). 24227320 10.1007/BF02010479

[b46] Bao L.-J., Maruya K. A., Snyder S. A. & Zeng E. Y. China’s water pollution by persistent organic pollutants. Environ. Pollut. 163, 100–108 (2012). 22325437 10.1016/j.envpol.2011.12.022

[b47] Yin G. . Chlorinated and brominated organic pollutants in shellfish from the Yellow Sea and East China Sea. Environ. Sci. Pollut. Res. 22, 1713–1722 (2015). 10.1007/s11356-014-3198-8 PMC6684575 24958534

[b48] Vetter W., Haase-Aschoff P., Rosenfelder N., Komarova T. & Mueller J. F. Determination of halogenated natural products in passive samplers deployed along the Great Barrier Reef, Queensland/Australia. Environ. Sci. Technol. 43, 6131–6137 (2009). 19746703 10.1021/es900928m

[b49] Li X. . Photodegradation of 2,2′,4,4′-tetrabromodiphenyl ether in nonionic surfactant solutions. Chemosphere 73, 1594–1601 (2008). 18842284 10.1016/j.chemosphere.2008.08.031

[b50] Zhang F., Lu G., Liu J., Yan Z. & Zhang Z. Bioaccumulation, distribution and metabolism of BDE-153 in the freshwater fish *Carassius auratus* after dietary exposure. Ecotox. Environ. Safe. 108, 16–22 (2014). 10.1016/j.ecoenv.2014.06.030 25038267

[b51] Yang S., Wu R. S. S., Mok H. O. L., Zhang Z. P. & Kong R. Y. C. Identification of a novel cytochrome P450 cDNA, CYP97E1, from the marine diatom *Skeletonema costatum* Bacillariophyceae. J. Phycol. 39, 555–560 (2003).

[b52] Munschy C., Héas-Moisan K., Tixier C., Pacepavicius G. & Alaee M. Dietary exposure of juvenile common sole (*Solea solea* L.) to polybrominated diphenyl ethers (PBDEs): Part 2. Formation, bioaccumulation and elimination of hydroxylated metabolites. Environ. Pollut. 158 (2010). 10.1016/j.envpol.2010.08.021 20864231

[b53] Liu F. . Multi-species comparison of the mechanism of biotransformation of MeO-BDEs to OH-BDEs in fish. Aquat. Toxicol. 114–115, 182–188 (2012). 10.1016/j.aquatox.2012.02.024 22446830

[b54] Guillard R. L. Culture of phytoplankton for feeding marine invertebrates. In Culture of marine invertebrate animals (eds Walter L. Smith & Matoira H. Chanley) 29–60 (Springer US, 1975).

[b55] Okumura Y., Koyama J., Takaku H. & Satoh H. Influence of organic solvents on the growth of marine microalgae. Arch. environ. con. tox. 41, 123–128 (2001). 10.1007/s002440010229 11462135

[b56] Wang H.-S. . Hydroxylated and methoxylated polybrominated diphenyl ethers in blood plasma of humans in Hong Kong. Environ. Int. 47, 66–72 (2012). 22771521 10.1016/j.envint.2012.06.004

[b57] Ho K.-L. . Urinary bromophenol glucuronide and sulfate conjugates: Potential human exposure molecular markers for polybrominated diphenyl ethers. Chemosphere 133, 6–12 (2015). 25817024 10.1016/j.chemosphere.2015.03.003

[b58] Mackay D. & Fraser A. Bioaccumulation of persistent organic chemicals: Mechanisms and models. Environ. Pollut. 110, 375–391 (2000). 15092817 10.1016/s0269-7491(00)00162-7

[b59] Mhadhbi L., Fumega J. & Beiras R. Toxicological effects of three polybromodiphenyl ethers (BDE-47, BDE-99 and BDE-154) on growth of marine algae *Isochrysis galbana*. Water Air Soil Pollut. 223, 4007–4016 (2012).

[b60] Källqvist T., Grung M. & Tollefsen K.-E. Chronic toxicity of 2,4,2′,4′-tetrabromodiphenyl ether on the marine alga *Skeletonema costatum* and the crustacean *Daphnia magna*. Environ. Toxicol. Chem. 25, 1657–1662 (2006). 16764486 10.1897/05-424r.1

